# Artificial intelligence in the diagnosis and management of dysphagia: a scoping review

**DOI:** 10.1590/2317-1782/e20240305en

**Published:** 2025-08-08

**Authors:** Rayane Délcia da Silva, Suzanne Bettega Almeida, Flávio Magno Gonçalves, Bianca Simone Zeigelboim, José Stechman-Neto, Angela Graciela Deliga Schroder, Weslania Viviane Nascimento, Rosane Sampaio Santos, Cristiano Miranda de Araujo

**Affiliations:** 1 Universidade Tuiuti do Paraná – UTP - Curitiba (PR), Brasil.; 2 Núcleo de Estudos Avançados em Revisão Sistemática e Meta-análise – NARSM, Universidade Tuiuti do Paraná – UTP - Curitiba (PR), Brasil.; 3 Núcleo de Inteligência Artificial em Saúde – NIAS, Universidade Tuiuti do Paraná – UTP - Curitiba (PR), Brasil.; 4 Instituto de Estudos de Reabilitação em Fonoaudiologia - Mataró, España.

**Keywords:** Artificial Intelligence, Machine Learning, Deep Learning, Deglutition, Deglutition Disorder, Inteligência Artificial, Aprendizado de Máquina, Aprendizado Profundo, Deglutição, Distúrbios da Deglutição

## Abstract

**Purpose:**

This scoping review aimed to map and synthesize evidence on technological advancements using Artificial Intelligence in the diagnosis and management of dysphagia. We followed the PRISMA guidelines and those of the Joanna Briggs Institute, focusing on research about technological innovations in dysphagia.

**Research strategies:**

The protocol was registered on the Open Science Framework platform. The databases consulted included EMBASE, Latin American and Caribbean Health Sciences Literature (LILACS), Livivo, PubMed/Medline, Scopus, Cochrane Library, Web of Science, and grey literature.

**Selection criteria:**

The acronym 'PCC' was used to consider the eligibility of studies for this review.

**Data analysis:**

After removing duplicates, 56 articles were initially selected. A subsequent update resulted in 205 articles, of which 61 were included after applying the selection criteria.

**Results:**

Videofluoroscopy of swallowing was used as the reference examination in most studies. Regarding the underlying diseases present in the patients who participated in the studies, there was a predominance of various neurological conditions. The algorithms used varied across the categories of Machine Learning, Deep Learning, and Computer Vision, with a predominance in the use of Deep Learning.

**Conclusion:**

Technological advancements in artificial intelligence for the diagnosis and management of dysphagia have been mapped, highlighting the predominance and applicability of Deep Learning in examinations such as videofluoroscopy. The findings suggest significant potential to improve diagnostic accuracy and clinical management effectiveness, particularly in neurological patients. Identified research gaps require further investigations to solidify the clinical applicability and impact of these technologies.

## RESUMO

**Objetivo:**

Esta revisão de escopo visou mapear e sintetizar evidências sobre avanços tecnológicos utilizando Inteligência Artificial no diagnóstico e manejo da disfagia. Seguimos as diretrizes PRISMA e do Instituto Joanna Briggs, com foco em pesquisas sobre inovações tecnológicas em disfagia.

**Estratégia de pesquisa:**

O protocolo foi registrado na plataforma Open Science Framework. As bases de dados consultadas incluíram EMBASE, Literatura Latino-Americana e do Caribe em Ciências da Saúde (LILACS), Livivo, PubMed/Medline, Scopus, Cochrane Library, Web of Science e literatura cinzenta.

**Critérios de seleção:**

O acrônimo 'PCC' foi utilizado para considerar a elegibilidade dos estudos para esta revisão.

**Análise de dados:**

Após a remoção de duplicatas, 56 artigos foram inicialmente selecionados. Uma atualização posterior resultou em 205 artigos, dos quais 61 foram incluídos após a aplicação dos critérios de seleção para análise qualitativa.

**Resultados:**

A videofluoroscopia da deglutição foi utilizada como exame de referência na maioria dos estudos. Em relação às doenças de base presentes nos pacientes que participaram dos estudos, houve predominância de diversas condições neurológicas. Os algoritmos utilizados variaram entre as categorias de Machine Learning, Deep Learning e Visão Computacional, com predominância do uso de Deep Learning.

**Conclusão:**

Os avanços tecnológicos em inteligência artificial para o diagnóstico e manejo da disfagia foram mapeados, destacando a predominância e aplicabilidade do Deep Learning em exames como a videofluoroscopia. Os resultados sugerem um potencial significativo para melhorar a precisão diagnóstica e a eficácia do manejo clínico, especialmente em pacientes neurológicos. As lacunas em pesquisa apontadas necessitam de investigações futuras para solidificar a aplicabilidade clínica e o impacto dessas tecnologias.

## INTRODUCTION

Dysphagia, a symptom that impairs swallowing and can lead to pulmonary complications, dehydration, and malnutrition, is a growing concern in studies due to its impact on patients' quality of life and the healthcare system. It affects about 12-13% of hospitalized patients, rising to 30% in the elderly, contributing to a 47.5% increase in hospitalizations in this group, and is considered a geriatric syndrome. The prevalence can be as high as 60% in intensive care or home nursing settings, with rates varying based on associated comorbidities^(1,2)^.

Distinguishing the etiology and performing early and accurate diagnosis play a fundamental role in the prognosis of dysphagia, which is why they have been the subject of extensive research. Evaluation modalities are generally divided between clinical approaches and imaging examinations, which complement each other. However, these assessments are considered subjective, and some examinations may face accessibility issues or lack standardized protocols. Additionally, special attention must be given to the risk-benefit aspects for the patient, making it essential for this assessment to be evidence-based^(3,4)^.

Artificial Intelligence (AI) consists of a set of technologies designed to perform tasks in a manner similar to human intelligence. Intelligent agents are trained using data until they can carry out their functions autonomously. Subfields of AI include Machine Learning (ML) algorithms, which identify patterns and make predictions, and Deep Learning (DL), which is considered more complex due to its use of layered neural networks. These technologies contribute to the emergence of new hypotheses, discoveries, and task optimization in healthcare, aiming for a safer and more efficient approach^(5-8)^. With technological advancements in healthcare, artificial intelligence plays a significant role, particularly in image analysis. In the context of dysphagia, AI offers new perspectives for identifying swallowing alterations and facilitating the rehabilitation process. Therefore, this review aims to map and synthesize evidence regarding technological advancements with AI in the diagnosis and management of dysphagia.

## METHODS

This comprehensive review was conducted in accordance with the guidelines of the Preferred Reporting Items for Systematic Reviews and Meta-Analyses Extension for Scoping Reviews (PRISMA-ScR) and the recommendations for scoping reviews by the Joanna Briggs Institute^(9)^. It was registered on the Open Science Framework (OSF) platform^(10)^.

### Eligibility criteria

The acronym 'PCC' was used to formulate the following research question: “What is the evidence regarding technological advancements involving artificial intelligence in the diagnosis and management of dysphagia?” This acronym was also applied to determine the eligibility criteria for studies included in this review, representing:

- **P** = Population (Humans of any age group);
- **C** = Concept (Use of Artificial Intelligence);
- **C** = Context (Aid in the treatment and diagnosis of dysphagia).

#### Inclusion criteria

To map studies with a higher level of evidence, only primary and analytical studies were included, such as clinical trials, cohorts, case-control studies, cross-sectional, prospective, or retrospective studies, which used AI in the evaluation or treatment of dysphagia. There were no restrictions regarding the gender, ethnicity of individuals, language of studies, publication date, and diagnosis.

#### Exclusion criteria

The following exclusion criteria were applied: a) animal studies; b) studies without any use of technology and/or innovation involving AI; c) studies without dysphagia management; d) reviews, case reports, case series, personal opinions, letters, posters, and conference abstracts.

### Information sources and search

Word combinations were adapted for each of the seven selected electronic databases as sources for the search, namely: EMBASE, Latin American and Caribbean Health Sciences Literature (LILACS), LIVIVO, PubMed/Medline, Scopus, Cochrane Library, and Web of Science. Additionally, grey literature was also used as a source of information through AshaWire, Google Scholar (100 most relevant results), and ProQuest Dissertations & Theses Global (Appendix A).

Searches in electronic databases and grey literature were conducted on October 27, 2022, and an update was performed on November 3, 2023. All references were managed, and all duplicate studies were removed using appropriate software (EndNote® X7 Thomson Reuters, Philadelphia, PA). The reference lists of all included articles were checked using the web application Citation Chaser^(11)^, searching for both the citations used by these studies and the articles that cited them.

### Selection of sources of evidence

Article selection was carried out in two phases. In the first phase, two reviewers (R.D.S and S.B) independently reviewed the titles and abstracts of all references. All articles that did not meet the pre-established criteria were excluded at this stage. In the second phase, the same reviewers independently read the full text of the articles selected in the first phase. When there was no consensus even after discussion, a third reviewer (R.S) was involved for the final decision.

To facilitate independent reading, the Rayyan website^(12)^ was used. In addition to the two reviewers who conducted blind assessments, a third team member (C.A) acted as a moderator.

### Data charting process and data items

The collected data consisted of study characteristics (author, year of publication), population characteristics (age and pathology), algorithms and AI techniques used, model evaluation metrics, and outcomes.

If the necessary data were incomplete, efforts were made to contact the authors to obtain unpublished data. Authors could be contacted via email for three consecutive weeks in search of more information.

All relevant information was extracted and mapped, with extraction performed by the two main reviewers, followed by final data verification using the Bing AI tool^(13)^. As this is a descriptive review, any measures of effect were considered and used in the qualitative synthesis.

### Reporting bias

To reduce the likelihood of reporting bias, a comprehensive search strategy was conducted through seven electronic databases, including a non-English language database (LILACS). Additionally, a search of grey literature was also conducted to check for the existence of studies meeting eligibility criteria but not yet published.

## RESULTS

### Selection of sources of evidence

**Figure 1:**
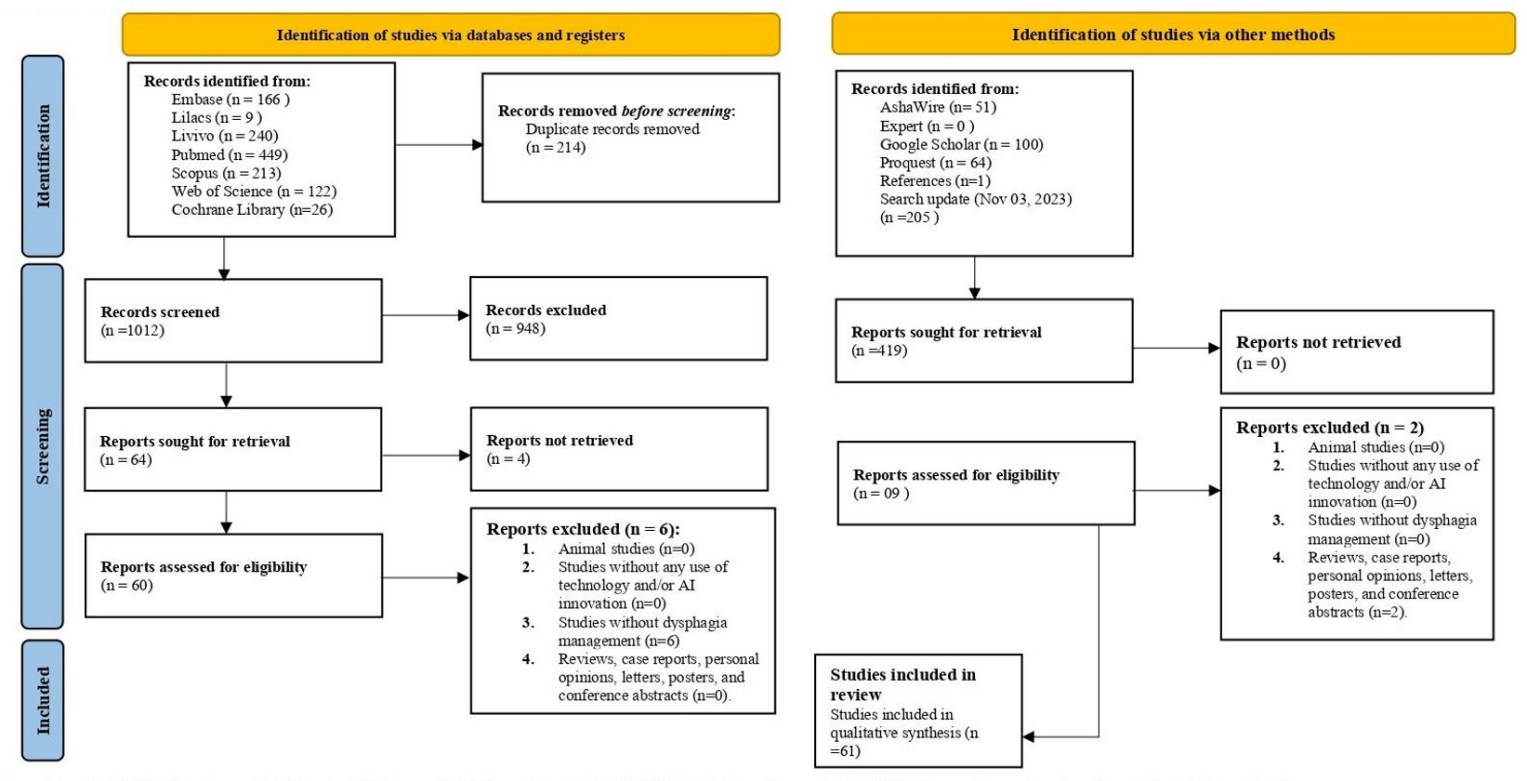
Literature search flowchart and selection criteria

Source: Page et al.(14)

The flow of studies through the scoping review process is presented in Figure 1. A total of 1.225 articles were retrieved from seven electronic databases. After removing duplicates, 1.012 references remained. Subsequently, 948 studies were excluded based on eligibility criteria. Four articles could not be located even after contacting the authors. A search of grey literature, reference lists, and an update of the databases on November 3, 2023, were also conducted, resulting in 69 studies for full-text reading. After the full-text review (second phase), 8 articles were excluded (see Appendix B). Based on the established inclusion criteria, 61 studies were identified as suitable for qualitative synthesis and results mapping.

### Characteristics of sources of evidence

The included studies were published from 1999^(15)^ to 2023^(16-19)^. The sample sizes of the studies ranged from one^(18)^ to 3408^(16)^ participants, with ages ranging from ten months^(20)^ to 94^(21,22)^ years. Most studies utilized some form of clinical evaluation with imaging or sound examination as a comparator in the analyses or as an objective to enhance the examination for diagnosis. Videofluoroscopy swallowing study (VFSS) was utilized in studies^(16,23-28)^, with four of them concurrently using high-resolution manometry^(27,29-31)^, only two studies^(32,33)^ used fiberoptic endoscopic evaluation of swallowing (FEES), and 2 studies reported electromyography use^(3,17)^. Sound resources as an auxiliary method in evaluation were also used^(17,21,25,26,28,34-46)^. Only one study focusing on therapeutic biofeedback and without information on associated examination methodology was found^(47)^.

**Figure 2:**
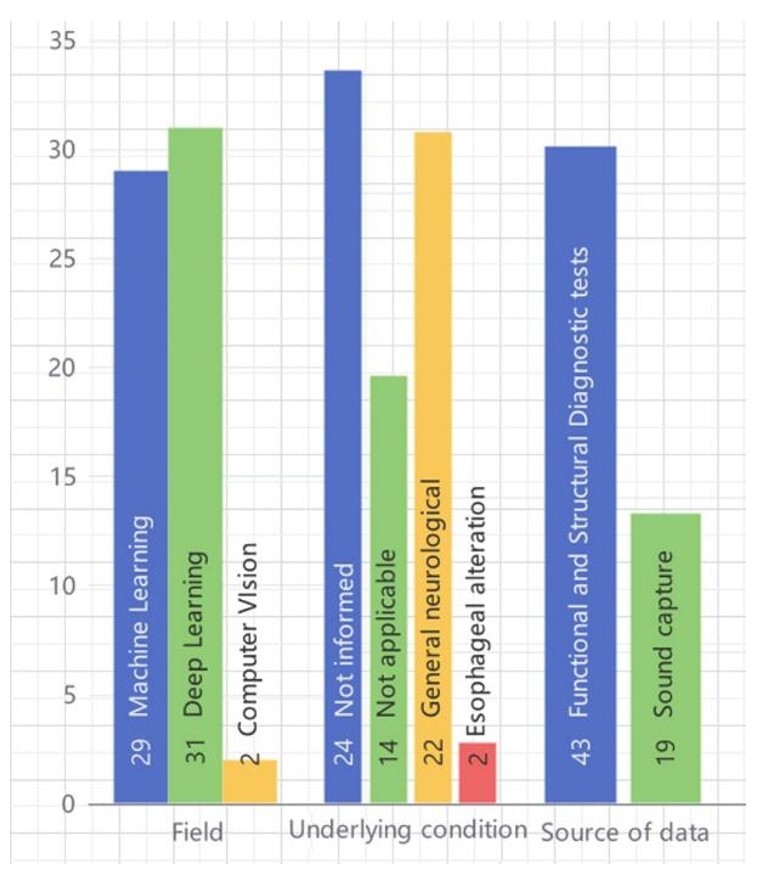
Number of studies according to field, underlying condition, and data source

Regarding the underlying diseases present in the patients participating in the studies, there is a predominance of various neurological diseases, with stroke being the most cited in 12 studies^(16,18,21,22,27,32,36,40,46,48-51)^, neurodegenerative diseases like Parkinson's were present in 3 studies^(22,24,27)^, and two studies mentioned esophageal alterations^(42,52)^. Many studies did not report the population's pathology or had no applicability due to the research methodology. The algorithms used varied within the classification of Machine Learning^(2,3,16,20,25-28,30,32,35,37,39,40,42,44,45,48-50,53-62)^, Deep Learning^(17-19,21-24,29,31,33,34,36,38,41,43,46,51,52,57,63-75)^, and Computer Vision^(15,47)^ (Figure 2). Several studies have reported high accuracy in using AI and machine learning techniques for dysphagia assessment. For instance, deep learning models like U-Net and CNNs have achieved performance metrics such as F1 scores exceeding 0.9 and accuracy rates of 97.8%, indicating their robustness in detecting swallowing events and anatomical structures. Other methods, including support vector machines (SVM) and Mask-RCNN, have demonstrated high accuracy in classifying swallowing events, with metrics like sensitivity and specificity reaching over 90%. These findings emphasize the potential of AI-driven tools in improving diagnostic accuracy for dysphagia^(51,68,69,75)^.

Despite the considered effective results, all highlighted the need for further studies in the area. Descriptive characteristics of all included studies were recorded in Appendix C.

### Results of individual sources of evidence

Studies on AI in dysphagia primarily rely on imaging resources such as VFSS for comparative analysis due to its high reliability^(16,23-28)^. However, the images generated by the examination are still analyzed by human judgment^(38,61,63,65)^. Since the swallowing process is considered complex, each structure contributes uniquely, with the hyoid bone being one of the most studied^(21,22,50)^. VFSS, along with high-resolution manometry, has also been considered in the evaluation of pharyngeal and esophageal anatomical structures^(27,29-31)^, and in the use of electromyography, AI aims to improve signal capture and analysis quality^(3,17)^.

Evaluation using sound resources is also part of the research, considered a safe, practical, and non-invasive support, and besides assisting in evaluation, it can be used as a biofeedback therapeutic resource. Cervical auscultation, commonly used in clinical evaluation, now consists of a range of digital resources such as accelerometers, microphones, and sensors that facilitate the analysis of specific parameters. Increasingly used in research practices, they enable diagnostic clinical markers and specific analyses^(20,26,28,32,35-37,39,41-44,53,56,57,66)^.

In research, the most addressed pathologies in adults were predominantly related to the neurological area, with stroke being highlighted in several studies^(16,21,22,24,27,36,40,46,48-51)^. In the pediatric population, cerebral palsy was the most cited condition in studies focusing on this age group^(20,37,49)^. The algorithms used in the studies varied according to the needs of each research, but most of them were classified between Machine Learning and Deep Learning, with significant accuracy levels.

## DISCUSSION

The integration of AI in healthcare can enhance professionals' efficiency by optimizing data management and influencing decisions^(8)^. When combined with imaging resources for real-time swallowing evaluation, it becomes possible to offer more accurate diagnoses and improve therapeutic planning for patients with dysphagia. Key studies demonstrate high performance of deep learning models, such as CNNs and Mask-RCNN, in detecting and segmenting bolus movements in VFSS with precision metrics exceeding 90% in certain frameworks (Appendix C). This highlights the potential of AI not just in diagnostics but also in automating labor-intensive aspects of analysis^(65,69)^. It was observed that most studies focus on adults and use VFSS as a reference for reliability. Neurological diseases are frequently mentioned as the primary underlying conditions, and a variety of algorithms classified as ML or DL demonstrate good performance in achieving their goals. Stroke-related dysphagia, for example, has been widely studied with algorithms like SVMs and deep neural networks demonstrating robust accuracy in predicting aspiration events and laryngeal vestibule closure^(29,32)^. This focus underscores the significant burden that neurological conditions place on clinical resources and the need for innovations to improve workflow efficiency.

A videofluoroscopic swallowing study (VFSS), considered the reference examination in swallowing assessment, is frequently cited in research. However, its use presents challenges due to radiation exposure and limited availability in some locations. Additionally, the lack of a standardized protocol and variability in training, when provided, as well as in interpretations, directly impacts diagnostic accuracy. Recent methodologies integrating VFSS with AI-powered models have shown promise in addressing these limitations, such as high-resolution segmentation of swallowing structures via Mask-RCNN achieving intersection-over-union scores of 0.71^(65)^. VFSS is used by many professionals involved in dysphagia assessment and rehabilitation as the primary tool. FEES, another reference examination, is mentioned less frequently but faces similar challenges regarding availability and patient discomfort. Although both VFSS and FEES have high sensitivity and specificity, the need for human interpretation in defining results raises questions and inspires possibilities for creating algorithms that can automate evaluation and contribute to the analysis of specific structures^(4,5,32,57,66)^. Thus, AI contributes by aiming to automate and standardize some identification and recognition processes in an objective and effective manner. The same approach applies to the assessment of the esophageal region, which is also being studied. High-resolution manometry, considered highly accurate for this anatomical area, allows for the diagnosis of esophageal motor disorders. Additionally, studies utilizing deep learning and neural network classifiers for esophageal motility have reported sensitivity metrics above 85%, offering promising diagnostic complements^(29,30)^ (Appendix C).

In addition to these technologies, the biomechanics of swallowing is extremely complex, offering various forms of interpretation and analysis. The swallowing process involves not only images but also vibrations and sounds generated by the anatomical structures. Digital tools, such as accelerometers and high-resolution cervical auscultation sensors, have also shown significant diagnostic potential, with accuracy levels reaching 98% in distinguishing safe from unsafe swallows^(57)^ (Appendix C). However, these methods often rely on imaging examinations to validate accuracy, as cervical auscultation can be affected by technical interferences and the experience of the evaluator. Despite these advancements, challenges remain regarding the generalizability of these tools across different patient populations and clinical environments. Despite these advances, challenges remain regarding the generalizability of these tools across different patient populations and clinical environments^(76)^.

The algorithms used in the research, which achieved satisfactory levels in evaluation metrics with varied results, belong to two interrelated fields of AI that play a significant role in machine learning and data-driven decision-making. Machine Learning involves identifying patterns in data, making predictions, classifying information, and making decisions based on available information. It focuses on developing algorithms and models that enable systems to “learn”. Deep Learning, on the other hand, is a subcategory of Machine Learning, distinguished by its use of deeper neural networks. This distinction is particularly relevant in tasks involving large volumes of unstructured data, such as images, audio, and text, with audio and images being the most common data types in studies^(77,78)^.

The integration of artificial intelligence in the evaluation and treatment of dysphagia holds great potential to enhance diagnostic accuracy and professional efficiency. Traditional methods, such as VFSS and FEES, face challenges related to availability and human interpretation. Machine learning and deep learning algorithms offer solutions to standardize and automate assessments, making them more objective. Research must progress to overcome the limitations of traditional methods, improving dysphagia management and patients' quality of life.

## CONCLUSION

In conclusion, this study aimed to map and synthesize evidence on the integration of artificial intelligence in the diagnosis and management of dysphagia. The findings demonstrate that AI, particularly through machine learning and deep learning algorithms, offers transformative potential by improving diagnostic accuracy, standardizing evaluations, and addressing limitations of traditional methods such as VFSS and FEES. AI technologies have shown high performance in tasks like bolus movement detection, esophageal motility analysis, and the interpretation of biomechanical signals, contributing to more objective and efficient clinical workflows. However, challenges such as limited generalizability, the need for standardized protocols, and variability in clinical settings remain significant barriers to widespread adoption. The study underscores the importance of further research to validate these technologies across diverse populations and clinical environments. Addressing these gaps is essential to ensuring the ethical and effective integration of AI into routine clinical practice, ultimately enhancing the quality of care for patients with dysphagia.

## Appendix A Database search strategy

**Table:**

**Database**	**Search**
**Lilacs**	(“inteligência artificial” OR “IA” OR “inteligência computacional” OR “inteligência de máquina” OR “aprendizagem de máquina” OR “aprendizagem profunda” OR “inteligencia artificial” OR “IA” OR “inteligencia computacional” OR “inteligencia de la máquina” OR “rede neuronal” OR “aprendizaje de la máquina” OR “aprendizaje profundo” OR “artificial intelligence” OR “AI” OR “computational intelligence” OR “machine Intelligence” OR “neural network” OR “machine learning” OR “deep learning”)
	AND (“Trastornos de la deglución” OR “Trastorno de la deglución” OR “trastorno de deglución” OR “trastornos de deglución” OR “Disfagia” OR “Disfagia orofaríngea” OR “Disfagia esofágica” OR “Transtornos da Deglutição” OR “Transtorno da Deglutição” OR “Disfagia” OR “Disfagia esofágica” OR “Deglutition Disorders” OR “Deglutition Disorder” OR “Swallowing Disorders” OR “Swallowing Disorder” OR “Dysphagia” OR “Oropharyngeal Dysphagia” OR “Esophageal Dysphagia”)
**PubMed**	(“artificial intelligence”[MeSH Terms] OR “AI” OR “computational intelligence” OR “machine Intelligence” OR “neural network” OR “machine learning” OR “machine learning”[MeSH Terms] OR “deep learning”[MeSH Terms] OR “deep learning”)
	(“Deglutition Disorders”[MeSH Terms] OR “Deglutition Disorders” OR “Deglutition Disorder” OR “Swallowing Disorders” OR “Swallowing Disorder” OR “Dysphagia” OR “Oropharyngeal Dysphagia” OR “Esophageal Dysphagia”)
	#1 AND #2
**SCOPUS**	(“artificial intelligence” OR “AI” OR “computational intelligence” OR “machine Intelligence” OR “neural network” OR “machine learning” OR “machine learning” OR “deep learning” OR “deep learning”) AND (“Deglutition Disorders” OR “Deglutition Disorders” OR “Deglutition Disorder” OR “Swallowing Disorders” OR “Swallowing Disorder” OR “Dysphagia” OR “Oropharyngeal Dysphagia” OR “Esophageal Dysphagia”)
**Web of Science**	1. TS=(“artificial intelligence” OR “AI” OR “computational intelligence” OR “machine Intelligence” OR “neural network” OR “machine learning” OR “machine learning” OR “deep learning” OR “deep learning”)
	2. TS=(“Deglutition Disorders” OR “Deglutition Disorders” OR “Deglutition Disorder” OR “Swallowing Disorders” OR “Swallowing Disorder” OR “Dysphagia” OR “Oropharyngeal Dysphagia” OR “Esophageal Dysphagia”)
	3. #1 AND #2
**Embase**	('artificial intelligence' OR 'AI' OR 'computational intelligence' OR 'machine Intelligence' OR 'neural network' OR 'machine learning' OR 'machine learning' OR 'deep learning' OR 'deep learning') AND ('Deglutition Disorders' OR 'Deglutition Disorders' OR 'Deglutition Disorder' OR 'Swallowing Disorders' OR 'Swallowing Disorder' OR 'Dysphagia' OR 'Oropharyngeal Dysphagia' OR 'Esophageal Dysphagia')
**Livivo**	(“artificial intelligence” OR “AI” OR “computational intelligence” OR “machine Intelligence” OR “neural network” OR “machine learning” OR “machine learning” OR “deep learning” OR “deep learning”) AND (“Deglutition Disorders” OR “Deglutition Disorders” OR “Deglutition Disorder” OR “Swallowing Disorders” OR “Swallowing Disorder” OR “Dysphagia” OR “Oropharyngeal Dysphagia” OR “Esophageal Dysphagia”)
**Cochrane Library**	(“artificial intelligence” OR “AI” OR “computational intelligence” OR “machine Intelligence” OR “neural network” OR “machine learning” OR “machine learning” OR “deep learning” OR “deep learning”) AND TITLE-ABS-KEY (“Deglutition Disorders” OR “Deglutition Disorders” OR “Deglutition Disorder” OR “Swallowing Disorders” OR “Swallowing Disorder” OR “Dysphagia” OR “Oropharyngeal Dysphagia” OR “Esophageal Dysphagia”)
**AshaWire**	(“artificial intelligence” OR “AI” OR “computational intelligence” OR “machine Intelligence” OR “neural network” OR “machine learning” OR “machine learning” OR “deep learning” OR “deep learning”) AND (“Deglutition Disorders” OR “Deglutition Disorders” OR “Deglutition Disorder” OR “Swallowing Disorders” OR “Swallowing Disorder” OR “Dysphagia” OR “Oropharyngeal Dysphagia” OR “Esophageal Dysphagia”)
**Google Scholar**	“artificial intelligence” AND (deglutition OR Dysphagia) filetype:pdf
**ProQuest**	NOFT(“artificial intelligence” OR “AI” OR “computational intelligence” OR “machine Intelligence” OR “neural network” OR “machine learning” OR “machine learning” OR “deep learning” OR “deep learning”) AND NOFT(“Deglutition Disorders” OR “Deglutition Disorders” OR “Deglutition Disorder” OR “Swallowing Disorders” OR “Swallowing Disorder” OR “Dysphagia” OR “Oropharyngeal Dysphagia” OR “Esophageal Dysphagia”)

## Appendix B Reasons for study exclusion

**Table:**

**Author, Year**	**Reason for Exclusion**
Crary M, Sanchez J, Carnaby-Mann G, Carvajal P, Sura L, Lin S, Rampersad A. 2011	2
Dean J, Wong K, Gay H, Welsh L, Jones AB, Schick U, Oh JH, Apte A, Newbold K, Bhide S, Harrington K, Deasy J, Nutting C, Gulliford S. 2018	3
Lee SJ. 2020	3
Lee WH. 2021	3
Matsuda Y, Ito E, Kuroda M, Araki K. 2022	3
Mayo CS, Mierzwa M, Moran JM, Matuszak MM, Wilkie J, Sun G, Yao J, Weyburn G, Anderson CJ, Owen D, Rao A. 2020	3
Ursino S, Giuliano A, Martino FD, Cocuzza P, Molinari A, Stefanelli A, Giusti P, Aringhieri G, Morganti R, Neri E, Traino C, Paiar F. 2021	3
Ryu Y. Kim JH, Hyun J, Kim TU,Kim S, Lee SJ. 2023	2

Legends:

1 Animal studies;

2 Studies without any use of technology and/or innovation involving AI;

3 Studies without dysphagia management;

4 Reviews, case reports, case series, personal opinions, letters, posters, and conference abstracts

REFERENCE

1. Crary M, Sanchez J, Carnaby-Mann G, Carvajal P, Sura L, Lin S, Rampersad A. Accuracy of computer algorithms in the identification of swallows by acoustic signal. Dysphagia Research Society; 2011.

2. Dean J, Wong K, Gay H, Welsh L, Jones AB, Schick U, Oh JH, Apte A, Newbold K, Bhide S, Harrington K, Deasy J, Nutting C, Gulliford S. Incorporating spatial dose metrics in machine learning-based normal tissue complication probability (NTCP) models of severe acute dysphagia resulting from head and neck radiotherapy. Clin Transl Radiat Oncol. 2018 Jan;8:27-39.

3. Lee SJ. Application of Artificial Intelligence in the Area of Dysphagia. J Korean Dysphagia Soc. 2020;4-9.

4. Lee WH. Evaluation and Management of Dysphagia Based on Digital Health Technologies. J Korean Dysphagia Soc. 2021;(2):105-110.

5. Matsuda Y, Ito E, Kuroda M, Araki K. A Basic Study for Predicting Dysphagia in Panoramic X-ray Images Using Artificial Intelligence (AI)-Part 1: Determining Evaluation Factors and Cutoff Levels. Int J Environ Res Public Health. 2022 Apr 9;19(8):4529.

6. Mayo CS, Mierzwa M, Moran JM, Matuszak MM, Wilkie J, Sun G, Yao J, Weyburn G, Anderson CJ, Owen D, Rao A. Combination of a Big Data Analytics Resource System With an Artificial Intelligence Algorithm to Identify Clinically Actionable Radiation Dose Thresholds for Dysphagia in Head and Neck Patients. Adv Radiat Oncol. 2020 Jan 12;5(6):1296-1304.

7. Ursino S, Giuliano A, Martino FD, Cocuzza P, Molinari A, Stefanelli A, Giusti P, Aringhieri G, Morganti R, Neri E, Traino C, Paiar F. Incorporating dose-volume histogram parameters of swallowing organs at risk in a videofluoroscopy-based predictive model of radiation-induced dysphagia after head and neck cancer intensity-modulated radiation therapy. Strahlenther Onkol. 2021 Mar;197(3):209-218.

## Appendix C Characteristics of included studies

**Table:**

**AUTHORS**	**EVALUATION METHOD**	**SAMPLE SIZE (AVERAGE AGE)**	**UNDERLYING DISEASE**	**TECHNIQUE (ALGORITHM)**	**RESULTS**	**METRIC**	**CONCLUSION**
Ariji et al.^(68)^	Images from VFFS were continuously converted into 15 static images per second using deep learning.	12(20±89)	N/I	Deep Learning (Neural network U-Net)	The results showed high performance values, exceeding 0.9 for both test datasets.	The performance metrics used were the Jaccard index (JI), the Sørensen-Dice coefficient (DSC), and sensitivity.	Using a deep learning segmentation method in artificial intelligence, we automatically segmented the areas of food bolus in the VFFS images; This model also allowed for the assessment of aspiration and laryngeal invasion.
Bandini et al.^(69)^	The methodology involved training a machine learning model using a database of videofluoroscopic swallowing studies, as well as testing and validating the model.	78 (44.7 ± 17.9y)	N/A	Deep Learning (Convolutional Neural Networks - CNNs)	The study achieved an F1 score exceeding 0.9 and correlations with reference trajectories exceeding 0.9, indicating promising results for the effectiveness of the framework in VFSS analysis.	They used the F1 score and Pearson correlation coefficient (ry) to evaluate performance.	The authors conclude that the use of artificial intelligence in this context can save time and resources while providing reliable and consistent results.
Basiri et al.^(42)^	To classify normal swallowing and dysphagia, a Support Vector Machine (SVM) was used, where the system is trained and tested using the leave-one-out approach.	22	Gastroesophageal Reflux Disease. Machine Learning (Support Vector Machine - SVM).	Machine Learning (Support Vector Machine - SVM).	They managed to improve the quality of the signals, especially those mixed with unwanted noise.	Signal accuracy of 66.1% and subject accuracy of 95.7%.	Swallowing sound analysis can be useful in detecting dysphagia in patients with Gastroesophageal Reflux Disease.
Merey et al.^(37)^	An accelerometer system was used to capture movement data during swallowing in children, and classification algorithms were applied to quantify and classify swallowing characteristics compared to VFFS.	29 (6.8 ± 4.8y)	Cerebral Palsy, seizure disorder, developmental delay, brain injury, and Down Syndrome.	Machine Learning (Support Vector Machine - SVM, with a Radial Basis Function - RBF kernel).	The obtained result was a mean adjusted accuracy of 89.6% ± 0.9 for discriminating between safe and unsafe swallows in children with neurogenic dysphagia.	The metric used to evaluate classifier performance was the adjusted accuracy.	Accelerometry can be an effective approach for quantitative classification of pediatric swallowing.
Chang et al.^(15)^	Usage of a knowledge-based snake algorithm to track the movement of pharyngeal bolus in VFFS images.	1	N/A	Computer Vision (K-SNAKE)	The results indicated that the K-SNAKE algorithm is accurate and efficient, with average differences in boundary identification of 1.29 mm for lateral images and 2.13 mm for antero-posterior images. The algorithm also demonstrated faster processing times and higher reproducibility compared to manual tracing methods.	The algorithm's performance was measured in terms of efficiency, reproducibility, and accuracy.	The knowledge-based snake algorithm can be applied accurately and efficiently to track the movement of pharyngeal bolus.
Coyle and Sejdić^(26)^	Utilization of data science methods to analyze high-resolution cervical auscultation signals compared to VFSS.	354	N/I	Machine Learning (Deep Neural Network - DNN)	The results indicate that these algorithms can differentiate between safe and unsafe swallows with a high degree of accuracy.	Reported performance metrics include sensitivity and specificity.	It was concluded that data science offers new promising tools to address the issue of high-resolution cervical auscultation.
Cuadros-Acosta and Orozco-Duque^(3)^	The methodology involved the acquisition of sEMG data during swallowing, defining criteria to identify low-quality signals, and developing an automatic detection algorithm.	61 (43.4 ± 16.6 y)	N/A	Machine Learning (Random Forest)	Our results demonstrate how the three-stage scheme can automate the analysis of signal quality from a swallowing dataset obtained from patients diagnosed with dysphagia, implementing a random forest classifier that utilizes three features.recursos.	Accuracy of 98 ± 1.74%	The proposed scheme can be applied to improve existing segmentation methods by removing signals with a high noise rate, thus enhancing the quality analysis of sEMG signals during swallowing tasks.
Lee et al.^(22)^	A swallowing motion analysis software was used to obtain positional data of the hyoid bone.	77 (19 ± 94y)	Parkinson's Disease and Stroke Deep Learning (Multi-Domain Networks)	Deep Learning (Multi-Domain Networks)	The proposed method achieved high accuracy in tracking the hyoid bone, with a DSC of 0.92 for cervical vertebrae and 0.87 for the hyoid bone. The RMSE for the mean trajectory coordinates was 7.83 pixels.	The performance was evaluated using metrics such as Dice Coefficient (DSC) and Root Mean Square Error (RMSE).	The proposed algorithm can provide the capability to automatically analyze hyoid movements during swallowing in clinical practice and potentially enable decision-making regarding diagnostic and therapeutic modalities based on quantitative swallowing assessments.
Das et al.^(64)^	Swallowing acceleration signal collection and implementation of hybrid fuzzy logic neural networks.	28	N/I	Deep Learning (Convolutional Neural Networks)	Hybrid fuzzy logic neural networks showed satisfactory performance in detecting swallowing acceleration signals. FCN-1 Committee: correctly recognized 16 out of 16 artifact signals tested and correctly identified 31 out of 33 dysphagic swallowing signals. FCN-II correctly identified 24 out of 24 normal swallowing signals and 28 out of 29 artifact signals. Both showed no statistically significant difference between the actual (clinical) classification and the committee's classification.	Recognition Accuracy. Ambiguous Cases. Reliability.	The use of hybrid fuzzy logic neural networks can be beneficial for the recognition of swallowing acceleration signals. Both neural network committees demonstrated effectiveness in classifying swallowing signals and artifacts, showing that these automated systems based on neural networks with hybrid fuzzy logic are reliable and have the potential for broader clinical use.
Donohue et al.^(25)^	The methodology involved recording high-resolution cervical auscultation signals during swallowing in individuals with neurodegenerative diseases.	20 (35±82 y)	Neurological Changes	Machine Learning (Logistic Regression and Decision Trees)	The results indicated statistically significant differences in swallowing kinematic measurements between patient groups. Additionally, machine learning algorithms were able to annotate swallowing kinematic events, such as opening and closing of the upper esophageal sphincter, closure of the laryngeal vestibule, reopening of the laryngeal vestibule, and hyoid bone displacement, with varied accuracies compared to measurements made by human judges.	99% accuracy, 100% sensitivity, and 99% specificity	The study's conclusion highlights the potential of HRCA in characterizing swallowing function in patients with neurodegenerative diseases and in other patient populations.
Donohue et al.^(57)^	Healthy participants underwent high-resolution cervical auscultation evaluation during swallowing. Kinematic data were collected and analyzed to establish reference values for different age groups.	70 (62.66 ± 14.8y)	Neurodegenerative diseases.	Deep Learning (Convolutional Recurrent Neural Network - CRNN with two convolutional layers).	The results suggest that high-resolution cervical auscultation (HRCA) can characterize swallowing function in patients with neurodegenerative disease.	Accuracy of 88.78%, sensitivity of 91.28%, and specificity of 86.83% for upper esophageal sphincter events. The relative overlap percentage (ROP) of SRNN for tracking hyoid bone displacement was approximately 44.6%.	Preliminary results indicated promising accuracy in annotating these kinematic measures, suggesting that HRCA can be used non-invasively and accurately to assist in swallowing assessment in healthy adults and in determining screening criteria for dysphagia.
Donohue et al.^(28)^	Analysis of HRCA signals from swallowing in both healthy individuals and those with neurodegenerative diseases using simultaneous VFFS with non-invasive cervical sensors as reference.	71 (39±87y)	Neurodegenerative Diseases	Machine Learning (Support Vector Machine, Naïve Bayes, Logistic Regression, and Decision Tree classifiers).	The results from the mixed linear model revealed that 22 HRCA signal features extracted from the microphone and triaxial accelerometer were statistically significant (p < 0.05) for predicting whether the swallows were from healthy individuals or from patients with neurodegenerative diseases.	Accuracy of 76%, sensitivity of 76%, and specificity of 77%.	HRCA signals can be used to differentiate between swallows of healthy individuals and those with neurodegenerative diseases. It is a useful method for screening dysphagia with the potential to be a diagnostic complement to instrumental swallowing assessments.
Egashira et al.^(59)^	The networks were trained based on the RR intervals of the heart rate (RRI) to automatically identify any temporary increase in heartbeats, possibly related to the act of swallowing.	10 (± 22y)	N/A	Deep Learning (Three-level hierarchical neural network - 3NN and Convolutional Neural Network - CNN).	A correlation between heart rate and swallowing was observed.	83.20%	The model with CNN was able to detect swallowing more accurately and automatically, with the possibility of discriminating between different types of foods.
Enz et al.^(32)^	Individuals affected by stroke underwent acoustic swallowing evaluation. The results were compared with FEES.	26 (64.9 ± 15.6y)	Stroke	Machine Learning (Decision Tree)	The Doppler sonar correctly identified tracheal aspiration with a sensitivity of 100% and specificity of 91%, demonstrating promising diagnostic accuracy.	sensitivity of 100.0% and a specificity of 91.0%	It was concluded that acoustic swallowing evaluation can be a precise and effective option for diagnosing dysphagia in stroke-affected patients, providing a less invasive and more accessible approach.
Zhang et al.^(74)^	An intelligent algorithm for swallowing event recognition is being developed, utilizing Nyquist plots as input for a Convolutional Neural Network (CNN).	20 (± 25y)	N/A	Deep Learning (Convolutional Neural Network - CNN, ResNet-50)	The overall recognition accuracy of swallowing events achieved by the algorithm is 97.8%. This high accuracy demonstrates the effectiveness of the CIPG method and the ResNet-50 algorithm in accurately classifying different types of swallowing events.	The algorithm's performance is evaluated using accuracy, which is a standard metric for classification tasks.	The study confirms the effectiveness and superiority of the detection technique.
Frakking et al.^(20)^	The methodology employed consisted of analyzing swallowing sounds recorded during VFFS in children through digital cervical auscultation using an algorithm.	41 (±10 months)	Congenital syndromes, neurological conditions, respiratory problems, anatomical anomalies, and other conditions.	Machine Learning (Support Vector Machine - SVM)	Consistent differences were observed in the time characteristics, power spectral density, and spectral sub-band centroids between aspiratory and normal swallowing sounds in children.	Overall Accuracy: 98% Sensitivity for Aspiration Detection: 89% Sensitivity for Normal Swallowing Detection: 100% Positive Predictive Value (PPV): 100% for normal swallows.	. The study demonstrates that spectral and temporal characteristics of swallowing sounds can be effective in distinguishing between normal and aspiratory swallows in children, using machine learning techniques.
Freed et al.^(47)^	Development of a prototype intelligent assistant using artificial intelligence and natural language processing techniques. Tests were conducted with dysphagia patients to evaluate the usability and effectiveness of the assistant.	N/I	N/A	Computer Vision	The feedback confirmed the potential benefit for patients and provided guidance on prioritizing which safe feeding strategies are most important to monitor.	The pilot data showed an RMS estimation error of 3.6 degrees for the algorithm's ability to estimate head angle, which is smaller than the intra-subject variability of 5.2 degrees for correctly performed chin tucks.	The study suggests that intelligent assistants can play an important role in supporting dysphagia patients at home, improving food safety and quality of life.
Fujinaka et al.^(70)^	A CNN trained with VFFS data was proposed to segment cervical intervertebral discs. The network's performance was evaluated using segmentation evaluation metrics.	58	N/I	Deep Learning (Convolutional neural network - CNN)	The CNN achieved promising results in segmenting cervical intervertebral discs, demonstrating high precision and accuracy.	The method's performance is evaluated using pixel-wise F-measure, and the highest F-measure achieved was 0.880 when specific pre-processing and post-processing techniques were applied.	The method's performance is evaluated using pixel-wise F-measure, and the highest F-measure achieved was 0.880 when specific pre-processing and post-processing techniques were applied.
Caliskan et al.^(65)^	The Mask-RCNN model is used to detect boluses in videofluoroscopic swallowing images.	30	N/I	Deep Learning (Mask-RCNN)	Using a Mask R-CNN detection method, bolus detection and segmentation were performed with an mAP of 0.49 and an overlap of 0.71.	The average precision (mAP) was 0.49 and the intersection over union (IoU) was 0.71 for the training data. For independent test data, an mAP of 0.42 was achieved.	The proposed method showed robust detection results that can help improve the speed and accuracy of a process in clinical decision-making.
Hashimoto et al.^(71)^	The deep transfer learning model was utilized using AlexNet and high-gamma band power to classify intracranial electrocorticogram (ECoG) data.	8 (27.8 ± 11.6y)	Epilepsy	Deep Learning (framework AlexNet42)	The study results demonstrated that AlexNet, pretrained with visually meaningful images, can effectively be used for transfer learning from visually nonsensical ECoG signal images to decode swallowing intention.	Accuracy 74.01%, sensitivity 82.51%, specificity 95.38%	It was concluded that classification using the AlexNet model can be used as an effective swallowing decoder with intracranial electrocorticogram.
Hoffman et al.^(30)^	Pattern recognition using an Artificial Neural Network (ANN) was performed to determine if the pharyngeal components of the MBSImP and the state of penetration/aspiration could be identified from the graph.	30 (68.0 ± 11.8)	N/I	Machine Learning (Artificial neural network – ANN)	A Receiver Operating Characteristic (ROC) analysis was conducted, resulting in areas under the curve (AUC) of 0.8912 for safe swallows, 0.8187 for aspiration, and 0.8014 for penetrative swallows. The results indicate that the ANN model demonstrates high accuracy in classifying swallows of dysphagic patients.	Accuracy 89.4 ± 2.4%	The authors concluded that classifying high-resolution manometry data according to videofluoroscopic parameters using pattern recognition is a promising approach for evaluating esophageal function.
Hoffman et al.^(29)^	An Artificial Neural Network (ANN) was evaluated for its ability to classify swallows as safe, penetration, or aspiration. Videofluoroscopic Swallow Study (VFSS) was used as a reference for comparison.	25 (69.4 ± 15.5y)	Etiologies of neurological origin.	Machine Learning (Artificial neural network - ANN)	Receiver Operating Characteristic (ROC) analysis showed an average classification accuracy of approximately 91%.	The area under the ROC curve ranged from 0.902 to 0.981, indicating a high level of accuracy in the classifications.	The classification models demonstrate high accuracy in categorizing swallows of dysphagic patients as safe or unsafe.
Iyer et al.^(72)^	Training a convolutional neural network, sequentially, to segment structures related to swallowing and chewing in computed tomography images.	243	Head and neck cancer	Deep Learning (Auto-segmentation ResNet-101, DeepLabV3+ using the Pytorch)	The results showed that the median values of DSC were 0.87 for the masseters, 0.80 for the medial pterygoid muscles, 0.81 for the larynx, and 0.69 for the constrictor muscle.	The primary metric used to evaluate the algorithm's performance was the Dice Similarity Coefficient (DSC).	The hypothesis was confirmed, showing that the ensemble models produced more stable results across all structures.
Jones et al.^(31)^	The methodology involved collecting high-resolution pharyngeal manometry data in patients with early to intermediate stages of Parkinson's disease. The data were analyzed using pattern recognition techniques to identify swallowing disorders.	62 (±68.7y)	Parkinson's disease	Machine Learning (Artificial neural network - ANN)	The result indicated a maximum classification rate of 82.3% for 2 cc swallows when all parameters were considered. The addition of variability-based parameters improved classification rates, and using only manometric parameters resulted in similar rates to using all parameters.	Classification rates, sensitivity, and specificity.	The study suggests that changes in pressure during swallowing may be sensitive indicators of swallowing function problems related to Parkinson's disease.
Inoue et al.^(39)^	The methodology involved collecting data on respiratory flow, laryngeal movement, and swallowing sounds, and using machine learning techniques to classify swallowing patterns.	192 (54 ± 32y)	N/I	Machine Learning (Support Vector Machine - SVM)	With results showing a sensitivity of 82.4% and a specificity of 86.0%, these findings indicate the effectiveness of the method in screening examinations for swallowing function.	Sensitivity and specificity.	Despite the limitations evidenced by the 20% of misclassifications, the approach has the potential to improve the assessment process of swallowing function, especially when used in conjunction with wearable sensors.
Khalifa et al.^(46)^	The study utilized Recurrent Convolutional Neural Networks (RNNs) to segment the opening of the Upper Esophageal Sphincter (UES) from cervical auscultation signals. The proposed method is based on recurrent convolutional neural networks to extract the dynamics of swallowing vibrations from swallowing signals and use them to infer the moments when the UES opens and closes during swallowing.	116 (62.7 ± 15.5y)	Stroke and other medical conditions unrelated to stroke.	Deep Learning (Recurrent Convolutional Neural Networks - RNNs)	The results indicated that the algorithm achieved an average accuracy of 90.93%, with similar values for sensitivity and specificity compared to human assessments. These results demonstrate the potential of high-resolution cervical auscultation as a non-invasive tool for assessing swallowing kinematics.	The main metrics used for evaluation were accuracy, sensitivity, and specificity.	The results provided substantial evidence that HRCA signals combined with a deep network architecture can be used to delineate important physiological events occurring during swallowing.
Khalifa et al.^(36)^	Utilization of deep learning in high-resolution cervical auscultation recordings.	3144	Stroke	Deep Learning (Deep neural networks -DNNs)	The algorithm demonstrated superior performance compared to existing algorithms and showed its generalization when tested on completely unseen swallows from a different population. It correctly identified about 95% of the swallowing segment in over 90% of the attempts.	The algorithm's performance was evaluated using detection accuracy, which exceeded 95%. It also achieved high sensitivity and specificity values, calculated over the entire dataset after removing visually unidentified parts of the recordings.	Deep learning on high-resolution cervical auscultation recordings can be a non-invasive approach to identify swallows.
Kim et al.^(38)^	Comparison between human assessment and machine learning algorithm.	49 (40± 80y)	N/I	Deep Learning (U-net Recurrent Convolutional Neural Network (RCNN) -RNNs)	The results indicated that the deep learning model achieved near-perfect intra-examiner reliability and substantial to moderate inter-examiner reliability, comparable to human examiners. The Positive Predictive Rate (PRR) and Negative Predictive Rate (NRR) of the model were both 100%, demonstrating its reliability in detecting laryngeal penetration or aspiration.	The metrics used to evaluate the model's performance were Cohen's kappa coefficient, Positive Reliability Rate (PRR), and Negative Reliability Rate (NRR).	Computerized analysis using a deep learning model can provide a reliable method for detecting the presence of laryngeal penetration or aspiration in VFSS images.
Kritas et al.^(27)^	Patients with dysphagia underwent swallowing tests with VFSS and high-resolution manometry, and the data were used to train an Artificial Neural Network (ANN). Clinical data and swallowing test results were combined to develop a predictive model.	179 (±66y)	Dementia,Stroke, Progressive neuromuscular diseases.	Machine Learning (Artificial neural networks - ANN)	The result indicated that the ANN model provided a superior prediction of aspiration risk compared to the IRD. The ANN model returned a value between 0.00 and 1.00, reflecting the degree of swallowing dysfunction and its potential to cause aspiration.	The key metric used was the Swallowing Risk Index (SRI).	The results suggest that artificial neural network modeling can be a useful tool in predicting the use of pattern recognition techniques and has the potential to simplify the clinical assessment of various metrics that collectively define the complex interaction of dysfunctional swallowing characteristics leading to aspiration. Our findings seem to correlate with relevant clinical sequelae such as aspiration, aspiration pneumonia, and hospitalization.
Kuramoto et al.^(67)^	The use of a convolutional neural network to monitor and detect swallowing duration in real-time, compared to VFSS.	192	Head and neck cancer, cerebral hemorrhage, stroke, ALS (Amyotrophic Lateral Sclerosis), Guillain-Barré syndrome, myasthenia gravis, progressive supranuclear palsy, and spinocerebellar degeneration.	Deep Learning Convolutional Neural Networks (CNNs)	The deep learning model achieved an accuracy of 97.3% on the validation set, which comprised 20% of the data.	The model's performance was evaluated using accuracy as the metric.	In comparison with VF images, we found that the swallowing duration from GOKURI represents the main swallowing reflex time.
Santoso et al.^(53)^	Extraction of acoustic features and classification using machine learning algorithms.	15	Ñ/A	Machine Learning (A decision tree, support vector machine - SVM and neural network trained with the scaled conjugate gradient - SCG).	The decision tree, SVM, and SCG neural network were able to detect swallowing clips from cough, speech, neck movement, and noise artifact clips.	The AUC results for the algorithms are 0.970 for the Decision Tree, 0.961 for the SVM, and 0.971 for the Neural Network	Machine learning algorithms are effective in automatically detecting swallowing events based on sound.
Lai et al.^(18)^	This study aimed to evaluate the classification performance of Transformer models and convolutional networks in identifying swallowing and non-swallowing tasks using video data.	65(±43.2y)	N/A	Deep Learning (Transformer Models: TimeSFormer and Video Vision Transformer (ViViT), Convolutional Neural Networks: SlowFast, X3D, and R(2+1)D2).	The result showed that the X3D model achieved good to excellent performance, with an F1 score of 0.920 and an adjusted F1 score of 0.8852.	The primary metric used for evaluation was the F1 score.	The results indicate that the X3D model showed the best performance, with good to excellent performance (F1-score: 0.920; adjusted F1-score: 0.885) in classifying swallowing and non-swallowing conditions using its default activation function.
Lee et al.^(51)^	The technique involves automatic segmentation of anatomical structures such as the thyroid cartilage and the vocal fold complex (TVC) using the Mask R-CNN convolutional neural network on VFSS swallowing study videos.	12 (± 45y)	Dementia / Ischemic Stroke and Hemorrhagic Stroke / Brain Tumor / Neuromyelitis Optica.	Deep Learning (Mask R-CNN)	The Mask R-CNN algorithm auto-segmented the thyroid cartilage and vocal fold complex (TVC) with an average IoU of 0.43 ± 0.19, indicating a considerable level of accuracy in the segmentation process. The recall rates for the auto-segmentation of TVC and C1 spinous processes were 86.8% and 99.8%, respectively. The actual displacement of the larynx measured was 35.1 mm.	The metric used to evaluate the algorithm's performance is Intersection over Union (IoU).	The results obtained suggest that the proposed method can be a promising tool for quantitatively and quickly determining laryngeal elevation in clinical settings.
Lee et al.^(45)^	The methodology involved the collection of data from multiple sensors during swallowing, training of artificial neural networks, and fusion of sensor data to segment swallowing.	17 (46.9 ± 23.8y)	N/A	Machine Learning (Artificial neural network – ANN)	The results indicated that the combination of all four signal sources achieved the highest average accuracy of 88.5% and adjusted accuracy of 89.6%.	Sensitivity, specificity, precision, and adjusted precision.	Concludes that the use of artificial neural networks and fusion of multiple sensors is an effective approach to segmenting swallowing, offering potential to improve the diagnosis and treatment of swallowing disorders.
Lee et al.^(50)^	Development of a detection system based on image analysis of VFSS and computational algorithms.	116 (± 66.5 y)	Stroke	Machine Learning (Support Vector Machine - SVM)	High sensitivity and specificity in detecting swallowing difficulties.	The metrics used for evaluation were accuracy, sensitivity, specificity, and area under the receiver operating characteristic curve (AUC). The study's result showed exceptional discrimination performance, with an AUC of 0.9269.	The proposed system can aid in the detection of swallowing problems.
Lee et al.^(60)^	The use of IPMC for throat movement detection and classification using AI algorithms.	N/I	N/I	Machine Learning (Support vector machine algorithm - SVM)	The self-powered IPMC sensor was able to distinguish different pressures exerted by throat movements. Based on the amplitude and velocity of throat movement, the optimized SVM model was able to recognize coughs, murmurs, swallows, and head movements with high accuracy of 95%.	Accuracy: 95%	The proposed throat sensor has revealed its potential to be used as a promising solution for smart healthcare devices, which can benefit many practical applications such as human-machine interactions, sports training, and rehabilitation.
Lee et al.^(48)^	Analysis of swallowing data post-VFSS in patients with ischemic stroke to identify predictors of swallowing recovery (6 months) in post-stroke dysphagia patients.	137 (±68.7y)	Stroke	Machine Learning (Bayesian Networks)	Survival analysis revealed that swallowing recovery at 6 months post-stroke varies significantly based on clinical and radiological factors.	Area Under the ROC Curve (AUC): 0.802 F1 Score: 0.9062 Matthews Correlation Coefficient: 0.575	Early dysphagia and bilateral lesions were significant prognostic factors for swallowing recovery at six months post-stroke. Using a Bayesian network model based on 10 clinical and radiological factors, the prediction of swallowing recovery was feasible. The importance of bilateral subcortical lesions as relevant prognostic factors for long-term recovery is highlighted. Future studies with larger cohorts and external validation are necessary to develop predictive models of post-stroke dysphagia applicable in clinical practice.
Lee et al.^(61)^	The methodology employed involved the use of machine learning techniques to automatically measure the response time of the pharyngeal swallowing reflex in VFSS studies.	27 (64.9 ± 15.7y)	Central nervous system disease or neuromuscular disease.	Deep Learning (3D Convolutional Network - I3D)	The study achieved an average success rate of 98.2% for the training set and 97.5% for the validation set in detecting the swallowing reflex. The average time error between the predicted detection and the actual onset point was 0.210 seconds, and at the endpoint was 0.056 seconds for the validation set.	The performance is evaluated using the detection F1 score and the time error of the onset and endpoint of the swallowing reflex.	This automated approach can provide more accurate and consistent results compared to traditional manual analyses.
Lee and Park^(24)^	The methodology employed was based on 3D convolutional neural networks, trained with augmented VFSS data, to detect the pharyngeal phase of swallowing.	144 (63.26 ± 16.37y)	Central nervous system disorders (such as stroke, Parkinson’s disease, etc.) Neuromuscular diseases Cancer Other conditions (aging, pneumonia, etc.)	Deep Learning (Inflated 3D Convolutional Neural Networks)	The I3D models achieved high accuracy, with the I3D-RGB model reaching an accuracy rate of 95.91% and the I3D-Joint model achieving 95.64% after 30 thousand training iterations.	The performance of the I3D models is evaluated using accuracy rates.	It is concluded that inflated 3D convolutional networks can be an effective approach for detecting the pharyngeal phase in videofluoroscopic swallowing studies.
Lee et al.^(34)^	It was used Transfer Learning with pre-trained CNNs to perform the recognition of the pharyngeal phase in VFFS videos.	54 (70.67 ± 14.73y)	N/I	Deep Learning (Convolutional neural network -CNN)	The proposed method achieved accurate and robust results in classifying the pharyngeal phase in unedited videofluoroscopy studies.	Accuracy: Achieved a precision of 93.20% (±1.25%). Sensitivity: Reported a sensitivity of 84.57% (±5.19%). Specificity: 94.36% (±1.21%). AUC: The area under the curve (AUC) was 0.8947 (±0.0269).	The use of Transfer Learning with CNNs has proven to be effective for the automatic recognition of the pharyngeal phase in unedited videofluoroscopic swallowing studies, potentially facilitating clinical analysis and the diagnosis of swallowing disorders.
Lee et al.^(23)^	The methodology used deep learning technology to develop a model for detecting airway invasion in VFSS (videofluoroscopic swallowing studies).	106	N/I	Deep Learning (deep convolutional neural network - DCNN)	The results showed a high accuracy rate in detecting airway invasion in videofluoroscopy using the proposed model.	Accuracy of 97.2% in classifying image frames and 93.2% in classifying video files.	It was concluded that deep learning technology is effective in the automatic detection of airway invasion in videofluoroscopy.
Lizana García^(54)^	The study proposes an automatic delineation method for VFFS image analysis.	N/I	N/I	Machine Learning (MiFOD (Minimum of Function for Object Detection)	The results indicate that the algorithm performs well, with an average computation time of 0.39 seconds per frame without motion strategies and 0.71 seconds per frame with motion strategies.	The metric used to evaluate the algorithm's performance is the computation time per frame, which varies depending on whether motion strategies are utilized or not.	The proposed automatic delineation can facilitate the analysis and interpretation of videofluoroscopic swallowing studies, saving time and effort.
Mao et al.^(21)^	A two-layer feedforward neural network was developed to identify these discrete sounds. The network was trained using the backpropagation algorithm. Another feedforward network with the same configuration and inputs was created to identify breathing segments.	7 (13 ± 30)	N/A	Machine Learning (Multilayer feed forward neural networks	Among the different multi-layer feedforward neural networks examined in this study, the networks with one input layer (36 inputs), one hidden layer (with 9 hidden neurons), and one output layer showed the best performance.	Accuracy: 91.7%	The proposed method can be used for automated extraction of swallowing sounds from respiratory sounds in both healthy individuals and those with dysphagia.
Mao et al.^(21)^	Data collection with non-invasive motion sensors (accelerometry) on the neck during VFFS in patients suspected of dysphagia.	65 (19± 94y)	Twenty-one participants (18.42%) had a history of stroke.	Deep Learning (Stacked Recurrent Neural Network - SRNN)	The result indicated that the tracking accuracy of the SRNN closely approached human evaluator judgment, with an overall mean ROP of 51.60% across all test groups. This suggests the feasibility of using sensor signals for non-invasive tracking of hyoid bone movement.	Relative Overlap Percentage (ROP)	The results indicate that it is feasible to track hyoid bone movement based on sensor signals, and this tracking is influenced by the patient's diagnosis. This suggests the potential of the sensor as a non-invasive screening tool for swallowing and hyoid bone movement tracking, but further investigations are needed to assess its diagnostic value.
Martin-Martinez et al.^(62)^	The researchers developed an AI model based on machine learning and used clinical and radiological data to train the model. They also implemented a risk management approach to improve diagnostic accuracy.	2809 (82.47 ± 9.33y)	Neurological and respiratory changes.	Machine Learning (Random Forest)	The linear model consists of 31 variables that showed statistical significance after bivariate analysis. Sensitivity is 94%; specificity is lower at 41.6% (This indicates that there may be false positives, i.e., patients who were incorrectly classified as having dysphagia).	Area under the ROC Curve (AUCROC): 0.840; Sensitivity: 0.940; Specificity: 0.416; Positive Predictive Value: 0.834; Negative Predictive Value: 0.690.	The system has proven to be a useful tool for identifying patients at risk and assisting clinicians in making informed decisions about diagnosis and treatment.
Miyagi et al.^(35)^	Collecting swallowing sounds and applying classification algorithms.	27 (21± 47y)	N/A	Machine Learning (Support Vector Machine - SVM).	The results showed that in a two-class scenario (normal subjects and dysphagic), the maximum F-measure was 78.9%. In a four-class scenario (normal subjects, mild, moderate, and severe dysphagic), the F-measure values for the classes were 65.6%, 53.1%, 51.1%, and 37.1%, respectively.	Maximum F-measure was 78.9%.	Support vector machines can be a useful tool for classifying dysphagic swallowing sounds, provided that ample datasets can be obtained.
O’Brien et al.^(40)^	The researchers collected data from wearable sensors in patients with dysphagia, applied machine learning techniques to analyze the data, and identified relevant biomarkers.	505 (^+^18)	Stroke	Machine Learning (Random Forest)	The sensory measures encoding coordination between breathing and swallowing were important features related to the presence and severity of dysphagia.	N/I	The authors concluded that machine learning techniques may be promising for non-invasive monitoring and diagnosis of dysphagia.
Wilhelm et al.^(63)^	Utilization of deep learning techniques for VFFS examinations.	107	N/I	Deep Learning (Recurrent convolutional network - LRCN).	The area under the ROC curve, which measures the classifier's diagnostic ability, was 0.89. This indicates the promising potential of the algorithm as a screening tool for dysphagia in Videofluoroscopic Swallow Studies (VFFS).	Accuracy of 85%	The proposed method shows promise in assisting with the diagnosis of swallowing disorders.
Park et al.^(16)^	The Gugging Swallowing Screen (GUSS), an early assessment tool for dysphagia, was used in all patients, and its predictive value was compared with the ML models. Videofluoroscopic swallowing studies (VFSS) were used to confirm aspiration.	3408 (67±73y)	Stroke	Machine Learning (ridge regression, lasso regression, elastic net, random forest, extreme gradient boosting, support vector machines, k-nearest neighbors, and naive Bayes).	The result indicated that the ridge regression model had a good balance between sensitivity and specificity in predicting the risk of aspiration.	Area Under the Receiver Operating Characteristic Curve (AUROC) of 0.81.	This study demonstrated that a machine learning-based screening model was not inferior to GUSS in predicting aspiration in hospitalized patients with acute stroke.
Prabhu et al.^(55)^	They evaluated neural network models using acceleration signals obtained during swallowing and coughing from a set of normal individuals and those with dysphagia.	N/I	N/I	Machine Learning (Neural Networks)	The Neural Network Model I recognized and distinguished patterns with 100% accuracy, and Model II classified pharyngeal swallowing patterns with 93% accuracy. These models automate the pattern recognition process, aiding in the diagnosis of dysphagia.	The parameters extracted from the acceleration signal include peak-to-peak amplitudes, slopes, average frequency, number of zero crossings, and mean power.	The application of neural networks showed potential for recognizing acceleration patterns during swallowing and coughing.
Roldan-Vasco et al.^(49)^	The methodology employed consisted of collecting speech samples from patients with functional oropharyngeal dysphagia, extracting acoustic features, and using machine learning algorithms to analyze speech dimensions.	92 (60.17 ± 11.93y)	Ischemic stroke Dementia Muscular dystrophy Spinocerebellar ataxia Motor neuron disease Multiple sclerosis Myasthenia gravis Neuropathy Cerebral palsy Inflammatory myopathy	Machine Learning (SVM: support vector machine; MLP: multilayer perceptron; RF: random forest; DT: decision tree).	An area under the curve (AUC) of 0.86 ± 0.10 and a sensitivity of 0.91 ± 0.12 were obtained for the individual analysis of speech dimensions. Furthermore, a voting ensemble combining multiple speech dimensions yielded improved performance, suggesting that complementary information from distinct feature sets extracted from speech signals under dysphagia conditions enhances the overall classification accuracy.	The metric used for optimization was the AUC – ROC.	It was concluded that the use of machine learning techniques may represent a promising approach to support the diagnosis and treatment of patients with functional oropharyngeal dysphagia in a non-invasive and cost-effective manner.
Wang et al.^(44)^	The methodology involved the analysis of throat signals using an adaptive boosting algorithm and the implementation of a dysphagia detection system based on this analysis.	226 (±50y)	N/I	Machine Learning (Adaptive Boosting -Adaboost)	The proposed system achieved a classification accuracy of 71.2%, with a sensitivity of 66.6% and specificity of 76%.	Performance is measured using accuracy, sensitivity, and specificity.	The study concludes that the proposed dysphagia detection system using speech signals acquired through bone conduction headphones is a feasible and low-cost solution for dysphagia detection.
Sabry et al.^(41)^	Utilization of high-resolution cervical auscultation signals (HRCA).	136	N/I	Deep Learning Convolutional recurrent neural network - CRNN)	Automated estimation of laryngeal vestibule closure duration was feasible using high-resolution cervical auscultation signals.	Accuracy of approximately 75%.	This study found that the analysis of HRCA signal using advanced machine learning techniques.
Shaheen et al.^(19)^	Bolus segmentation network from VFFS image data. The data was split into 75/25 training and validation sets, and a 4-fold cross-validation was performed.	80	N/I	Deep Learning (U-Net for automated segmentation).	The average result across the entire validation set was a Dice coefficient of 0.67.	The performance metric used was the Dice coefficient.	This study succeeded in developing a segmentation network with a wide range of image quality and patient series using a standard U-Net. Through various additional tests on the U-Net in the form of residual blocks, no significant improvement was observed, while increasing the number of trainable parameters. It seems that, unlike more complex networks, the challenge still lies in the data itself.
Surdea-Blaga et al.^(52)^	Various machine learning techniques were applied to develop an algorithm capable of automatically classifying esophageal motility disorders according to the Chicago Classification.	N/I	Esophageal symptoms	Deep Learning (Convolutional Neural Networks -CNNs)	The authors claim to have found a strong correlation between the automatic diagnoses made by the algorithm and the diagnoses made by human experts.	Top-1 accuracy and F1 score of 86%.	The study demonstrates the potential of using machine learning algorithms to improve the diagnosis of esophageal motility disorders.
Suzuki et al.^(43)^	The system uses an electronic stethoscope to capture swallowing sounds, and an artificial intelligence system for real-time analysis. .	20 (23.5 ± 1.6y)	N/A	Machine Learning (Adaptive Boosting Adaboost)	The study found that the value of the INDEX was significantly higher in men than in women and higher in the seated position than in the supine position. This suggests that the algorithm can effectively identify swallowing sounds, which could be useful for bedside screening of swallowing conditions in patients with dysphagia.	The metric used for analysis is a swallowing index (INDEX), calculated based on the number of target sound frames over the total frames in the auscultation section.	The use of an electronic stethoscope and an artificial intelligence system can provide an effective real-time assessment of swallowing disorders.
Cesarini et al.^(56)^	Gathering voice data and applying machine learning algorithms to identify dysphagia biomarkers.	106 (50y)	N/I	Machine Learning (Gaussian Kernel SVM or RBF).	Dysphagic patients have most of their speech energy in the low-frequency spectrum between 40 and 120 Hz (below the usual range) and a rougher spectrum (confirmed by RASTA-type filtering and empirical evaluation by listening to the recordings).	90%	Voice analysis based on machine learning can be an effective approach to detect dysphagia biomarkers. The biomarkers suggest a “rougher” voice in dysphagic patients.
Weng et al.^(33)^	The FEES-CAD segments the input FEES video and classifies penetration, aspiration, vallecular residue, and residue in the hypopharynx based on the segmented FEES video. We collected and annotated FEES videos to train the network and tested the performance of FEES-CAD using FEES videos.	239	N/I	Deep Learning Convolutional Neural Networks (CNNs)	The FEES-CAD achieved an average DSC of 98.6%, demonstrating expert-level accuracy in detecting aspiration and penetration in swallowing studies.	Performance is measured using the Dice similarity coefficient (DSC), among other metrics.	Comprehensive experiments across various classification tasks show that FEES-CAD is effective in analyzing FEES videos.
Zhang et al.^(17)^	In the practical demonstration, the created patch was applied with a CNN model trained for the recognition of eleven swallowing activities, three of which involved actual food digestion, while four mimicked abnormal swallowing movements.	5(22±27)	N/A	Deep Learning (Convolutional Neural Networks - CNNs)	The study presents a high-density surface electromyography (HD-sEMG) electrode array, designed for precise recognition of swallowing activities on complex epidermal surfaces.	accuracy: 80%.	Compared to a commercial Ag/AgCl electrode, this electrode exhibited much lower contact impedance in the sEMG frequency range of 1 to 1000 Hz and half the baseline noise with significant skin deformations. In practical demonstration, this patch was applied with a CNN model trained for the recognition of eleven swallowing activities, three of which involved actual food ingestion, while four mimicked abnormal swallowing movements. An average high classification accuracy of 80% was achieved, indicating the potential of this system for dysphagia diagnosis.
Zhang et al.^(75)^	The study employs deep learning techniques to develop a model capable of automatically identifying and annotating cervical vertebrae in videofluoroscopy images.	530 (64.83 ± 13.56y)	N/I	Deep Learning (Convolutional Neural Networks - CNNs)	The algorithm achieved high precision, with a mean distance error of 4.20 ± 5.54 pixels, which is comparable to the human inter-observer error of 4.35 ± 3.12 pixels. 93% of the predicted points were within five pixels of the annotated pixels in an independent dataset.	They used the mean distance between predicted points and annotations as the metric, comparing it with human inter-observer error for validation.	The conclusion drawn is that the deep learning-based approach is effective and promising for automatically detecting anatomical points of interest in swallowing videofluoroscopy images with high precision.
Zhao et al.^(2)^	Speech analysis is conducted by extracting acoustic features from the voice, while throat vibration analysis is performed using accelerometer sensors placed on the throat.	N/I	N/A	Machine Learning (Support Vector Machine -SVM, Multi-Layer Perceptron - MLP and Adaptive Boosting -Adaboost)	The results demonstrate that the proposed system achieves a high accuracy rate in detecting dysphagia compared to traditional methods. The classification accuracy reaches up to 72.09%.	The performance is evaluated using accuracy, sensitivity, and specificity.	Since this model was established based on speech data collected from older adults, it may perform poorly on young patients with dysphagia because some speech characteristics change with age.

Caption: N/I = Not Informed; NA = Not Applicable; VFSS = Videofluoroscopic Swallowing Study; SRNN = Stacked Recurrent Neural Network; ROP = Relative Overlap Percentage; SVM = Support Vector Machine; RBF = Radial Basis Function; HRCA = High-Resolution Cervical Auscultation; ANN = Artificial Neural Network; CNN = Convolutional Neural Network; LRCN = Long-Short Term Memory Recurrent Convolutional Network; MLP = Multilayer Perceptron; RF = Random Forest; DT = Decision Tree; CRNN = Convolutional Recurrent Neural Network; DCNN = Deep Convolutional Neural Network
